# Structural Quality and Magnetotransport Properties of Epitaxial Layers of the (Ga,Mn)(Bi,As) Dilute Magnetic Semiconductor

**DOI:** 10.3390/ma13235507

**Published:** 2020-12-03

**Authors:** Tomasz Andrearczyk, Khrystyna Levchenko, Janusz Sadowski, Jaroslaw Z. Domagala, Anna Kaleta, Piotr Dłużewski, Jerzy Wróbel, Tadeusz Figielski, Tadeusz Wosinski

**Affiliations:** 1 Institute of Physics, Polish Academy of Sciences, Aleja Lotnikow 32/46, PL-02668 Warsaw, Poland; andrea@ifpan.edu.pl (T.A.); khrystyna.levchenko@univie.ac.at (K.L.); sadow@ifpan.edu.pl (J.S.); domag@ifpan.edu.pl (J.Z.D.); kaleta@ifpan.edu.pl (A.K.); dluzew@ifpan.edu.pl (P.D.); wrobel@ifpan.edu.pl (J.W.); figiel@ifpan.edu.pl (T.F.); 2 Faculty of Physics, University of Vienna, 1090 Vienna, Austria; 3 Department of Physics and Electrical Engineering, Linnaeus University, SE-391 82 Kalmar, Sweden

**Keywords:** dilute magnetic semiconductors, molecular-beam epitaxy, interfaces, lattice mismatch, Curie temperature, anisotropic magnetoresistance, spin-orbit coupling, spintronics

## Abstract

Structural analysis of epitaxial layers of the (Ga,Mn)(Bi,As) quaternary dilute magnetic semiconductor (DMS), together with investigations of their magnetotransport properties, has been thoroughly performed. The obtained results are compared with those for the reference (Ga,Mn)As layers, grown under similar conditions, with the aim to reveal an impact of Bi incorporation on the properties of this DMS material. Incorporation of Bi into GaAs strongly enhances the spin-orbit coupling strength in this semiconductor, and the same has been expected for the (Ga,Mn)(Bi,As) alloy. In turn, importantly for specific spintronic applications, strong spin-orbit coupling in ferromagnetic systems opens a possibility of directly controlling the direction of magnetization by the electric current. Our investigations, performed with high-resolution X-ray diffractometry and transmission electron microscopy, demonstrate that the (Ga,Mn)(Bi,As) layers of high structural quality and smooth interfaces can be grown by means of the low-temperature molecular-beam epitaxy method, despite a large difference between the sizes of Bi and As atoms. Depending on the applied buffer layer, the DMS layers can be grown under either compressive or tensile misfit strain, which influences their magnetic properties. It is shown that even small 1% Bi content in the layers strongly affects their magnetoelectric properties, such as the coercive field and anisotropic magnetoresistance.

## 1. Introduction

Dilute incorporation of bismuth atoms into a GaAs semiconductor strongly reduces its energy gap as well as the temperature dependence of the energy gap [1,2], which is especially useful in optoelectronic applications [3,4,5]. In addition, partial replacement of As atoms by much heavier Bi atoms, which causes a large relativistic correction to the GaAs band structure, strongly enhances the spin-orbit coupling strength in the Ga(Bi,As) ternary alloy [6,7]. The latter effect substantially affects electronic properties of semiconductors. Nowadays, the spin-orbit coupling is of particular significance for development of spintronics technology, as it enables to effectively control the magnetization direction in small ferromagnetic systems by means of electric current.

Recently, we have grown and investigated epitaxial layers of dilute magnetic semiconductor (DMS) (Ga,Mn)As with a small percentage of Bi atoms incorporated at As sites. That crystalline quaternary alloy, (Ga,Mn)(Bi,As), displays slightly lower Curie temperature and strongly modified magnetoelectric properties, as compared to analogous material without the Bi content [8,9,10]. Incorporation of an atomic fraction of just 0.3% Bi into the (Ga,Mn)As layer results in distinct modification of the crystal valence band, as revealed from our modulation photoreflectance spectroscopy measurements, and in a significant increase in its magnetic coercivity [8]. Our latest study, performed by means of spatially resolved (with nanometer resolution) low-energy muon spin relaxation spectroscopy, evidenced a homogeneous ferromagnetic ordering below the Curie temperature in (Ga,Mn)(Bi,As) layers with 1% Bi content, similar to that in the reference (Ga,Mn)As layers [11]. In the present study, we investigate structural and magnetotransport properties of (Ga,Mn)(Bi,As) epitaxial layers as compared with those of (Ga,Mn)As layers obtained under similar conditions.

The large differences in atomic radii and electronegativities between As and Bi, which result in the weak Ga-Bi bonding energy and large miscibility gap, require highly non-equilibrium growth conditions, such as low-temperature molecular-beam epitaxy (LT-MBE), to obtain high-quality Ga(Bi,As) layers [4]. Even though the low MBE growth temperature enhances the solubility of Bi in GaAs, the incorporation of Bi at As sites is limited. Excess Bi typically accumulates as droplets segregate at the sample surface [12], but it can also lead to the formation of structural defects like Bi_Ga_ antisites, Bi pairs and small Bi clusters in the bulk of the layer [13]. Thus, while we have succeeded in the growth of high quality (Ga,Mn)As layers using the LT-MBE technique and precise optimization of the substrate temperature depending on the intentional Mn content [14], the growth of quaternary (Ga,Mn)(Bi,As) alloy becomes a challenge.

In this paper, we present comparative results of structural analysis obtained by means of high-resolution X-ray diffractometry (HR-XRD) and transmission electron microscopy (TEM) for the epitaxial layers of both alloys grown under the compressive misfit strain as well as under the tensile one. In addition, we report on the results of electrical transport measurements of those layers, which confirm substantial enhancement of the spin-orbit coupling strength in the layers as a result of Bi incorporation into the (Ga,Mn)As DMS.

## 2. Materials and Methods

(Ga,Mn)(Bi,As) layers of 50 nm thicknesses, with 6% Mn and 1% Bi contents, were grown using the low-temperature MBE technique at the temperature of approximately 230 °C. The layers were grown on either semi-insulating [001]-oriented GaAs substrate or on the same substrate covered with a 0.63 µm thick In_0.2_Ga_0.8_As buffer layer. Reference (Ga,Mn)As layers with 6% Mn content and 50 nm thicknesses were grown under similar conditions. The growth conditions were optimized to reduce the concentrations of As_Ga_ antisite and interstitial Mn defects in the DMS layers, as described in [14]. The layer thickness was verified by reflection high-energy electron diffraction (RHEED) intensity oscillations, which were visible during the growth of the whole 50 nm thick layer. Mn composition was determined with accuracy of 0.1% from a change in the (Ga,Mn)As growth rate with respect to that of LT-GaAs [15].

To improve the structural quality and magnetic properties of the layers, they were subjected, after the growth, to a low-temperature annealing treatment performed at the temperature of 180 °C in air during 50 h. It has been shown that annealing thin (Ga,Mn)As layers at temperatures below the growth temperature results mainly in the out-diffusion of Mn interstitials, which are responsible for the reduction in hole concentration and magnetic moment in the as-grown layers [16,17,18]. In turn, recent results obtained by Puustinen et al. [19] using Rutherford backscattering spectrometry gave no evidence for Bi diffusing out of Ga(Bi,As) layers during annealing at temperatures of up to 600 °C. The annealed samples were subjected to the secondary-ion mass spectrometry (SIMS) analysis to determine the in-depth composition of the consecutive layers in the investigated samples. The detailed results of SIMS analysis were presented in our previous paper [11]. The depth profiles confirm a uniform distribution of all the elements in the layers and the Mn content and the thickness of Mn-containing top DMS layers.

The HR-XRD characterization of the samples was carried out at room temperature using a high-resolution Philips X’Pert MRD diffractometer (Philips Analytical B.V., Almelo, The Nederlands) equipped with X-ray mirror, four bounce Ge(220) asymmetric monochromator and Ge(220) three bounce analyzer in triple-axis configuration [20]. The structural perfection, lattice parameters and misfit strain were determined from the measured 2 *θ*/*ω* scans and reciprocal lattice maps for the symmetrical 004 and asymmetrical −2–24 Bragg reflections of Cu K*α*_1_ radiation with the 1.5406 Å wavelength. The X-ray reciprocal lattice maps were obtained by recording series of 2 *θ*/*ω* scans around a Bragg node for specific reflection and sampling the disappearance of the X-ray intensity as it moves away from the node. The high-resolution TEM imaging of cross-sections across the sample interfaces, prepared with a focused ion beam (FIB), were performed with a JEOL JEM2000EX transmission electron microscope (JEOL Ltd., Tokyo, Japan).

Low-temperature magnetotransport properties of the DMS layers were investigated employing micro-Hall-bars prepared from the layers. The Hall bars, of 20 µm width and 50 µm distance between the voltage contacts, were fabricated by means of electron-beam lithography patterning and wet chemical etching. We assume an appearance of a single magnetic domain inside such a small Hall bar at low temperatures, at least for the (Ga,Mn)As layers grown on GaAs substrate [21]. Four-probe longitudinal resistance of the Hall bars was measured using a 0.5 µA sensing current and low-frequency lock-in technique.

## 3. Results and Discussion

### 3.1. X-ray Diffraction Results

**Figure 1:**
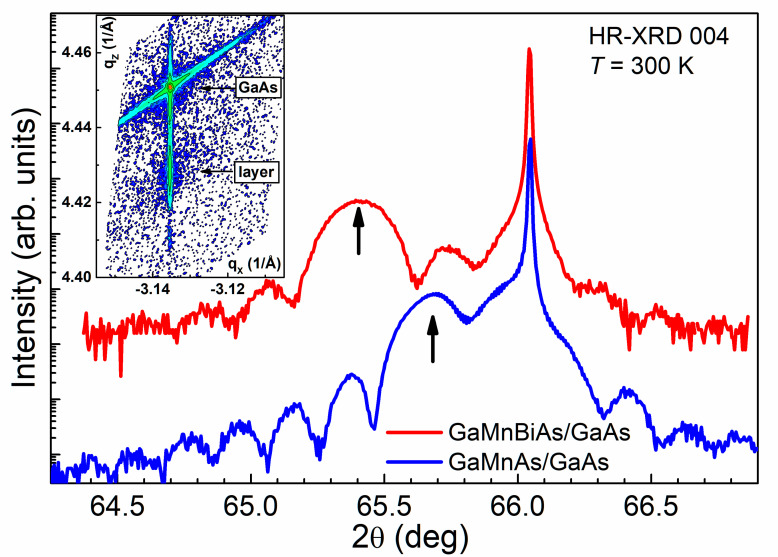
High-resolution X-ray diffraction spectra (2 *θ*/*ω* scans) for the 004 Bragg reflection for the (Ga,Mn)(Bi,As) and (Ga,Mn)As dilute magnetic semiconductor (DMS) layers epitaxially grown on (001) GaAs substrate. The narrow peaks correspond to the GaAs substrate and the broader ones at lower diffraction angles, indicated by the vertical arrows are reflections from the DMS layers. The spectra are vertically offset for clarity. The reciprocal lattice map of (Ga,Mn)As/GaAs sample for the −2–24 Bragg reflection, where the vertical and horizontal axes are along the out-of-plane [001] and in-plane [−1–10] crystallographic directions, respectively, in the 2 π/*d_hkl_* units, and where *d_hkl_* is the lattice spacing of corresponding crystallographic planes, is shown in the inset.

Figure 1 presents the HR-XRD diffraction spectra (2 *θ*/*ω* scans) for the symmetrical 004 Bragg reflection measured for both the (Ga,Mn)As and (Ga,Mn)(Bi,As) layers grown on GaAs substrate. The reciprocal lattice map (RLM) for the (Ga,Mn)As/GaAs sample, obtained for the asymmetrical −2–24 Bragg reflection, is shown in the inset in Figure 1. Here, the vertical axis, $q_{z}$, stands for the component of the reciprocal lattice vector perpendicular to the sample surface (parallel to the [001] crystallographic direction) and the horizontal axis, $q_{x}$, stands for the vector component parallel to the surface, along the [−1–10] direction. A similar RLM was obtained for the (Ga,Mn)(Bi,As)/GaAs sample. Vertical alignment of the nodes corresponding to the DMS layer and GaAs substrate implies the same in-plane lattice parameters of the layer and the substrate, evidencing the pseudomorphic growth of the layer subjected to elastic relaxation. The lower value of the $q_{z}$ component for the layer with respect to that of GaAs substrate indicates a larger perpendicular lattice parameter of the layer. These results are in agreement with the earlier findings [15,22], demonstrating that (Ga,Mn)As layers containing above 0.3% Mn, epitaxially grown on GaAs substrates, are subjected to biaxial compressive strain, which results from an increase in the lattice parameter of (Ga,Mn)As, proportional to the Mn content.

Accordingly, in the 004 diffraction spectra, shown in Figure 1, the broad peaks corresponding to reflections from the DMS layers appear at lower diffraction angles than the narrow ones corresponding to the GaAs substrate. Incorporation of 1% Bi into the (Ga,Mn)As layer causes a distinct shift of the corresponding peak to lower angles, indicating a significant increase in its lattice parameter perpendicular to the layer plane and an increase in the in-plane biaxial compressive strain. Well-defined interference fringes visible around the layer-related peaks imply homogeneous layer compositions and good interface quality. Mn content in the DMS layers and their thicknesses, calculated from the angular positions of the corresponding diffraction peaks and the angular spacing between the fringes, respectively, correspond rather well to the Mn content and layer thicknesses determined during the growth by the RHEED oscillations, as shown in Figure S1 in Supplementary Materials.

**Figure 2:**
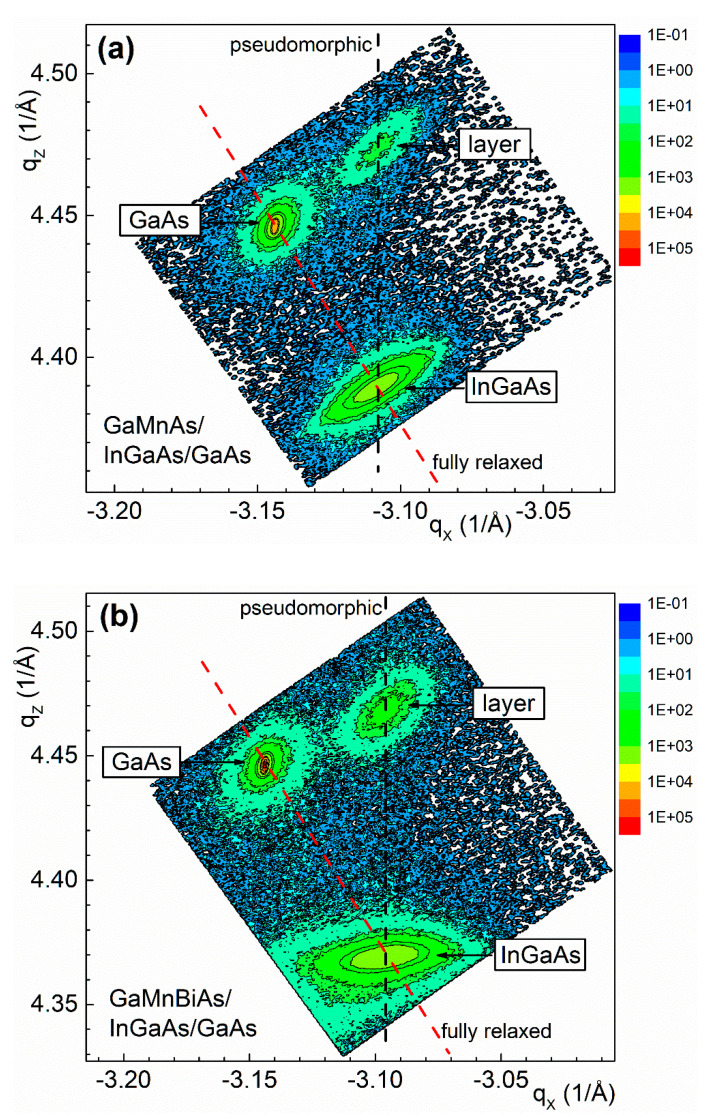
Reciprocal lattice maps of the (Ga,Mn)As/In_0.17_Ga_0.83_As/GaAs (**a**) and (Ga,Mn)(Bi,As)/In_0.20_Ga_0.80_As/GaAs (**b**) heterostructures for the −2–24 Bragg reflections. The vertical and diagonal dashed lines denote the reciprocal lattice map (RLM) node positions for pseudomorphic and fully relaxed layers, respectively.

The −2−24 reciprocal lattice maps for both the (Ga,Mn)As and (Ga,Mn)(Bi,As) layers grown on (In,Ga)As buffer layers are shown in Figure 2a,b, respectively, where the diffraction nodes for GaAs substrate, (In,Ga)As buffer, and DMS layers are visible. The vertical and diagonal dashed lines denote the RLM node positions for pseudomorphic (fully strained) and fully relaxed layers, respectively. For both heterostructures the thick (In,Ga)As buffer layers are fully plastically relaxed. On the other hand, both the (Ga,Mn)As and (Ga,Mn)(Bi,As) layers are pseudomorphically grown on the buffer under tensile misfit strain. In conclusion, the thicknesses of all the investigated DMS layers are below the critical thickness for plastic relaxation for the growth on GaAs substrate as well as on (In,Ga)As buffer, and the layers are fully strained under either the compressive biaxial strain or the tensile one, respectively.

Angular positions of the diffraction peaks for symmetrical and asymmetrical reflections are used to determine the out-of-plane, $a_{┴}$, and in-plane, $a_{\left|\right|}$, lattice parameters of the layers, respectively. The obtained $a_{┴}$ and $a_{\left|\right|}$ values are listed in Table 1.

The relaxed lattice parameters for the DMS epitaxial layers are calculated according to Equation (1),

(1) $$a_{rel}=\left(a_{┴}+2\frac{C_{12}}{C_{11}}a_{\left|\right|}\right)/\left(1+2\frac{C_{12}}{C_{11}}\right),$$

where *C*_11_ and *C*_12_ are the room-temperature elastic stiffness constants of the layers, which we assumed to be the same as those of GaAs [23], *C*_11_ = 118.4 GPa and *C*_12_ = 53.7 GPa. The calculated $a_{rel}$ parameters are listed in Table 1. In addition, the in-plane misfit strain (lattice mismatch) values in the layers, defined as $\varepsilon_{\left|\right|}=\left(a_{rel}-a_{\left|\right|}\right)/a_{\left|\right|}$, are calculated and are also listed in Table 1.

Misfit strain in (Ga,Mn)As layers strongly affects their magnetocrystalline properties. The layers grown under compressive misfit strain, with sufficiently large concentration of valence-band holes, exhibit in-plane magnetization with the easy axes along the in-plane [100] crystallographic directions at low temperatures [24,25]. On the other hand, the layers grown under tensile misfit strain display the easy magnetization axis along the out-of-plane [001] direction [26,27]. Qualitatively, the same magnetocrystalline anisotropy has been evidenced for the present (Ga,Mn)(Bi,As) layers by our recent investigations performed with magnetooptical Kerr effect (MOKE) magnetometry [28] and superconducting quantum interference device (SQUID, Cryogenic Consultancy Ltd., Cholsey, UK) magnetometry [11].

### 3.2. Transmission Electron Microscopy Results

**Figure 3:**
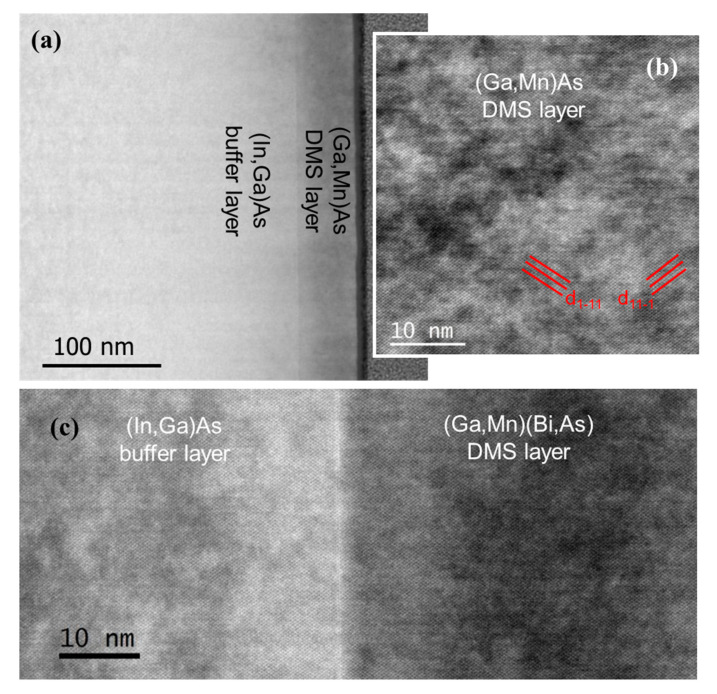
TEM images of the (In,Ga)As buffer layer/DMS layer interfaces in cross-section along the [110] zone axis for the (In,Ga)As/(Ga,Mn)As interface (**a**), where the (Ga,Mn)As layer under higher magnification is shown in (**b**), and for the (In,Ga)As/(Ga,Mn)(Bi,As) interface under higher magnification (**c**). Platinum cap layer protecting the TEM foil is seen at the far-right border in (**a**). Intersections of [111] crystallographic planes with the TEM foil are marked in (**b**).

**Figure 4:**
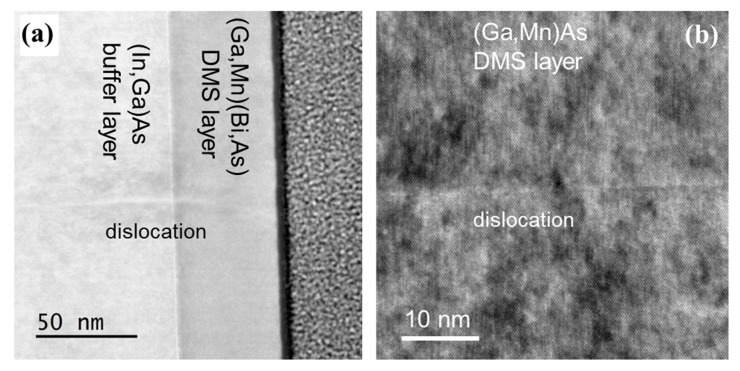
TEM images, under the same configuration as in Figure 3, showing threading dislocations intersecting the (In,Ga)As/(Ga,Mn)(Bi,As) interface (**a**) and, under higher magnification, in the (Ga,Mn)As layer grown on (In,Ga)As buffer (**b**).

Examples of cross-sectional TEM images of the (In,Ga)As buffer layer/DMS layer interfaces are shown in Figure 3 and Figure 4. All our TEM investigations performed for both the ternary and quaternary DMS layers grown on GaAs substrate as well as on (In,Ga)As buffers demonstrated rather high structural perfection of the DMS layers and smooth interfaces. {111} crystallographic planes are clearly visible in higher-resolution TEM images of the DMS layers (Figure 3b,c and Figure 4b) confirming their perfect zinc blend structure. Only a few structural defects were revealed in the TEM images of the DMS layers grown on (In,Ga)As buffers. Two examples of threading dislocations propagating across the (In,Ga)As buffer/(Ga,Mn)(Bi,As) interface and in the (Ga,Mn)As layer grown on (In,Ga)As buffer (visualized under higher resolution) are shown in Figure 4a,b, respectively.

Large lattice mismatch between the (In,Ga)As buffer layer and GaAs substrate is accommodated by the formation of high density of misfit dislocations at the interface. In general, misfit dislocations act as a source of threading dislocations, which propagate through the epitaxial layers during their growth, as demonstrated, e.g., in recent papers [29,30,31]. Some of those threading dislocations, which propagated through the whole (In,Ga)As buffer layer thickness and crossed the buffer layer/DMS layer interface, are revealed in Figure 4a,b.

### 3.3. Magnetotransport Properties

One of the most important parameters of ferromagnetic materials is their ferromagnetic Curie temperature, *T_C_*. Several methods can be used to estimate the *T_C_* values. Among them, electrical transport measurements belong to the most convenient ones, especially in a case of micro- or nanostructures patterned from thin DMS layers, for which standard magnetometry may be difficult due to the small volume of ferromagnetic material. Temperature dependence of the electrical resistivity of magnetic semiconductors displays a broad maximum around *T_C_* often used to estimate their Curie temperature. This maximum has been explained as resulting from magnetization-dependent spin-disorder scattering of charge carriers occurring near the paramagnetic–ferromagnetic phase transition [32,33,34]. Later on, it was demonstrated that for (Ga,Mn)As layers with a high carrier concentration (and rather high *T_C_*), the position of this maximum overestimates the *T_C_* value. Instead, the Curie temperature is more precisely determined from a maximum on the temperature derivative of resistivity vs. temperature dependence [35,36]. However, for (Ga,Mn)As layers with lower *T_C_* values of about 100 K and below, a number of experimental results, e.g., [35,37,38], show a different correlation: the temperature position of resistivity maximum corresponds better to the Curie temperature determined with the SQUID magnetometry than to that of the temperature derivative of resistivity.

**Figure 5:**
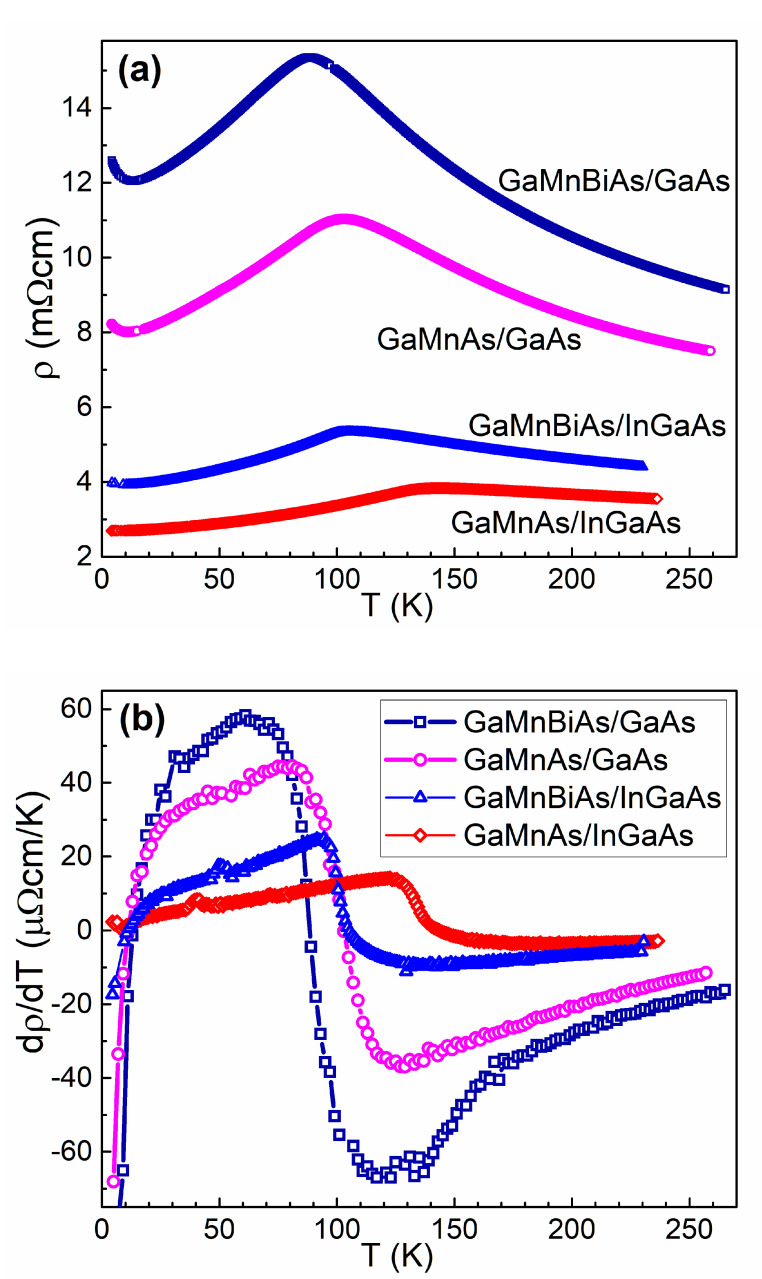
Temperature dependences of resistivity (**a**) and the derivative of resistivity with respect to temperature (**b**) for the (Ga,Mn)(Bi,As) and (Ga,Mn)As DMS layers grown on GaAs and on (In,Ga)As buffer.

Figure 5 shows the temperature dependences of resistivity (Figure 5a) and temperature derivative of resistivity (Figure 5b) obtained for the investigated DMS epitaxial layers under zero magnetic field in the temperature range from 4.2 K to about 270 K; the same were observed for both cooling down and heating up the samples. All the results display characteristic maxima at the temperatures listed in Table 2 together with the Curie temperature values obtained in our recent study by means of SQUID magnetometry and µSR spectroscopy [11]. For all the DMS layers investigated, of both the (Ga,Mn)As and (Ga,Mn)(Bi,As) alloys, the *T_C_* values estimated from resistivity vs. temperature dependence correspond quite well to the Curie temperatures determined using magnetometry methods. However, the values of maxima in the temperature derivative of resistivity of those layers underestimate the *T_C_* values by about 10 to 20 K. It is worth noting that the µSR spectroscopy measurements were performed for the DMS-layer contained wafers of about 1 cm^2^ area and the SQUID magnetometry measurements were done for smaller samples of the area about 0.15 cm^2^, cleaved from those wafers [11]. Furthermore, the present electrical resistivity measurements were carried out by employing much smaller Hall bars of about 1 × 10^−5^ cm^2^ area patterned on the same wafers. Noticeably different values of the Curie temperature estimations may result from different measurement methods used and/or from existing small inhomogeneities in the DMS epitaxial layers.

Higher resistivity of the (Ga,Mn)(Bi,As) layers, as compared with that of the (Ga,Mn)As reference ones, may result from the lower free hole concentration in the Bi-containing layers, previously revealed from our room-temperature Raman spectroscopy measurements performed for 10-nm thick layers [10], and/or lower hole mobility caused by increased carrier scattering at Bi-induced defects. Lower concentration of free holes, which are responsible for ferromagnetic ordering of Mn spins below *T_C_* in (Ga,Mn)As, may also be one of the reasons of lowering the Curie temperature caused by Bi incorporation into the (Ga,Mn)As layers.

Electrical transport in magnetic materials is strongly affected by their magnetic properties. In particular, the anisotropic magnetoresistance (AMR), which depends on the orientation of magnetization vector with respect to the electric current direction, occurs in the materials. For a thin ferromagnetic layer with the in-plane magnetization, containing a single magnetic domain, the AMR resistivity can be described by the relation [39]:

(2) $$\rho_{AMR}=\rho_{┴}-\left(\rho_{┴}-\rho_{\left|\right|}\right)\cos^{2}\theta ,$$

where $\rho_{┴}$ and $\rho_{\left|\right|}$ are the resistivities for in-plane magnetization vector oriented perpendicular and parallel to the current, respectively, and θ is the angle between the magnetization vector, *M*, and current, *I*, direction. In contrary to metallic ferromagnets, in which $\rho_{\left|\right|}$ is generally greater than $\rho_{┴}$ [39], in ferromagnetic (Ga,Mn)As, the opposite relation is valid, i.e., $\rho_{┴}$ > $\rho_{\left|\right|}$ [40]. The same also holds for (Ga,Mn)(Bi,As), as evidenced from our previous results [10].

**Figure 6:**
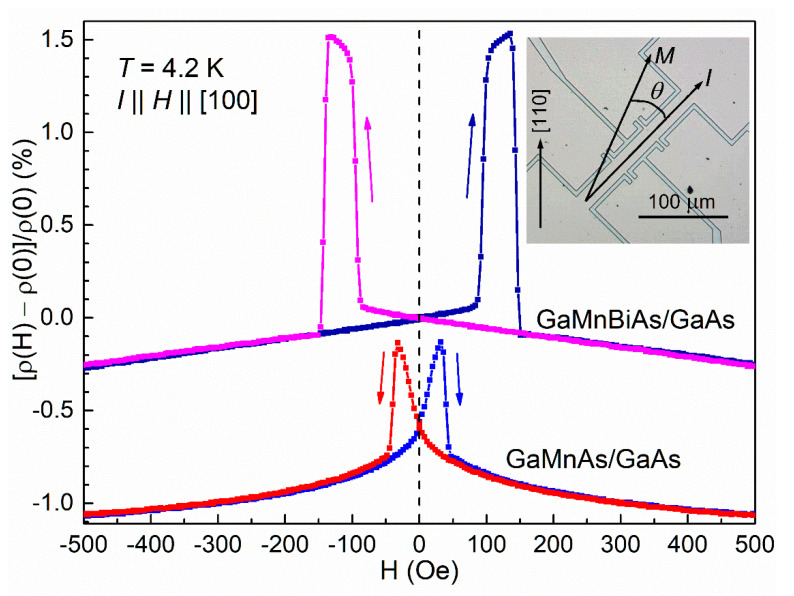
Magnetic-field-induced changes of resistivity normalized to the zero-field resistivity for the (Ga,Mn)(Bi,As) and (Ga,Mn)As layers grown on GaAs measured with micro-Hall-bars aligned along the in-plane [100] crystallographic direction, at the temperature of 4.2 K, while sweeping an in-plane magnetic field, parallel to the current, in opposite directions, as indicated by the arrows. The curves are vertically offset for clarity. Microscopic image of the micro-Hall-bar and the measurement configuration is shown in the inset.

Magnetoresistance measurements have been carried out on micro-Hall-bars, patterned from the DMS layers grown on GaAs under compressive misfit strain and displaying in-plane easy axes of magnetization. The measurements have been performed for various orientations of the in-plane magnetic field, *H*, at liquid helium temperature. Microscopic image of a micro-Hall-bar and the measurement configuration is shown in the inset in Figure 6. In order to compare AMR results for the (Ga,Mn)As and (Ga,Mn)(Bi,As) DMS layers we present, in Figure 6, the magnetic-field-induced changes of the layer resistivity normalized to that under zero magnetic field, [ρ(*H*) − ρ(0)]/ρ(0), measured with the Hall bars aligned along the in-plane [100] crystallographic direction under magnetic field applied parallel to that direction.

The up-and-down magnetic field sweep in the range of ±500 Oe results in nonmonotonic resistivity changes at low field, below the coercive values, superimposed on the isotropic negative magnetoresistivity extended to the higher field. Negative magnetoresistivity is a common property of conductive ferromagnetic materials caused by a reduction of spin-disorder scattering of charge carriers owing to alignment of magnetic ion spins in an external magnetic field. Moreover, the magnetic field suppresses the quantum interference contribution to the resistivity (the effect of weak localization), which is the main reason for a negative magnetoresistivity in (Ga,Mn)As at low temperatures, when the Mn spins are fully ferromagnetically ordered [41,42].

In the low field range, the up-and-down magnetic field sweep causes a rotation of the magnetization vector in the Hall bars by 360° between all the four in-plane [100] directions, corresponding to equivalent easy axes of magnetization of the DMS layers. While starting from a negative magnetic field, the magnetization vector takes consecutively the angles θ equal to 180°, 90°, 0°, 270° and again to 180°, resulting in four transitions of the AMR resistivities between their low and high values. As follows from Equation (2), the low values, occurring at θ equal to 180° and 0°, correspond to $\rho_{AMR}$ = $\rho_{\left|\right|}$, while the high values, occurring at θ equal to 90° and 270°, correspond to $\rho_{AMR}$ = $\rho_{┴}$. From the results presented in Figure 6 one can evaluate the magnitudes of AMR resistivity normalized to the zero-magnetic-field resistivity, ($\rho_{┴}$ − $\rho_{\left|\right|}$)/ρ(0), which are 0.5 × 10^−2^ and 1.5 × 10^−2^ for the (Ga,Mn)As and (Ga,Mn)(Bi,As) layers, respectively. The threefold increase in the AMR resistivity caused by an addition of 1% of Bi atoms into the (Ga,Mn)As layer results most probably from stronger spin-orbit coupling in the (Ga,Mn)(Bi,As) layer, making the latter material favorable for spintronic applications such as the magnetoelectric spin-orbit logic device recently proposed by Intel [43].

Sharp transitions of the AMR resistivities, observed in Figure 6 at magnetic fields of ±40 Oe and ±140 Oe for the (Ga,Mn)As and (Ga,Mn)(Bi,As) Hall bars, respectively, confirm an existence of single magnetic domains in the micro-Hall-bars of both the DMS layers. Those field values, corresponding to the coercive fields in the layers, show that Bi incorporation into (Ga,Mn)As layers results in a significant increase in their coercivities, as previously observed for thinner, 10 nm-thick layers [9]. Similar sharp jumps of the planar Hall resistance, observed for (Ga,Mn)As layers while sweeping magnetic field over the coercive field values, were attributed to nucleation and propagation of magnetic domain walls [44], occurring over a narrow magnetic field range, as shown for (Ga,Mn)As layers with in-plane magnetization by the direct magneto-optical domain imaging [21].

## 4. Conclusions

Epitaxial layers of quaternary dilute magnetic semiconductor (Ga,Mn)(Bi,As) and reference (Ga,Mn)As layers were grown using low-temperature molecular-beam epitaxy on either GaAs or (In,Ga)As buffer layers under different misfit strain conditions. Structural characterization of the layers was performed using high-resolution X-ray diffractometry and transmission electron microscopy. Structural analysis of the layers revealed that all of them were grown pseudomorphically on either GaAs or (In,Ga)As buffer under compressive and tensile misfit strain, respectively. Carefully optimized growth conditions and post-growth annealing treatment resulted in good crystalline quality of the layers with homogeneous compositions, well-defined and smooth interfaces and low concentrations of structural defects.

Ferromagnetic Curie temperatures of the layers evaluated from the maxima in resistivity vs. temperature dependences were in good agreement with those determined directly from magnetometry measurements. The *T_C_* values of (Ga,Mn)(Bi,As) layers were lower than those of the reference (Ga,Mn)As layers by about 15–30%. The (Ga,Mn)(Bi,As) layers were distinguished by larger coercive fields and much larger magnitudes of anisotropic magnetoresistivity. The latter likely resulted from an enhancement of the spin-orbit coupling strength caused by incorporation of a small amount of heavy Bi atoms into the (Ga,Mn)As layer. The increased spin-orbit coupling can be advantageous for developing novel functionalities, e.g., electrical control of magnetization direction through the spin-orbit torque effect, prospective for future spintronic device applications.

## Figures and Tables

**Table 1:** The out-of-plane and in-plane lattice parameters for the investigated DMS epitaxial layers grown on GaAs substrate and (In,Ga)As buffers. The calculated relaxed lattice parameters of the layers and in-plane misfit strain are also listed. The positive and negative values of the strain correspond to the compressive and tensile strain, respectively.

DMS Layer/Buffer	$a_{┴}\;(Å)\;(\pm 0.0001)$	$a_{\left|\right|}\;(Å)\;(\pm 0.0005)$	$a_{rel}\;(Å)$	$\varepsilon_{\left|\right|}\;(Å)\;(\times {10^{-}}^{4})$
GaMnAs/GaAs	5.6824	5.6538	5.6688	26.5
GaMnBiAs/GaAs	5.7032	5.6538	5.6797	45.8
GaMnAs/In_0.17_Ga_0.83_As	5.6220	5.7125	5.6650	−83.1
GaMnBiAs/In_0.20_Ga_0.80_As	5.6292	5.7355	5.6798	−97.2

**Table 2:** The Curie temperature values (with experimental errors) for the investigated DMS epitaxial layers grown on GaAs substrate and (In,Ga)As buffers obtained from our superconducting quantum interference device (SQUID) magnetometry and µSR spectroscopy measurements, reported in [11], and from the temperature values of resistivity maxima and those of temperature derivative of resistivity (present investigations).

DMS Layer/Buffer	*T_C_* (K) (±2) (SQUID)	*T_C_* (K) (±3) (µSR)	*T_C_* (K) (±1) (*ρ*(*T*)_max_)	*T_C_* (K) (±2) (*dρ*/*dT*(*T*)_max_)
GaMnAs/GaAs	105	100	103	81
GaMnBiAs/GaAs	90	85	88	63
GaMnAs/In_0.17_Ga_0.83_As	145	140	144	123
GaMnBiAs/In_0.20_Ga_0.80_As	100	100	105	94

## Supplementary Materials

The following are available online at https://www.mdpi.com/1996-1944/13/23/5507/s1, Figure S1: Simulation of high-resolution X-ray diffraction spectra for the 004 Bragg reflection for the (Ga,Mn)(Bi,As) and (Ga,Mn)As DMS layers epitaxially grown on (001) GaAs substrate.

## References

[1] Alberi K., Wu J., Walukiewicz W., Yu K.M., Dubon O.D., Watkins S.P., Wang C.X., Liu X., Cho Y.-J., Furdyna J. (2007). Valence band anticrossing in mismatched III-V semiconductor alloys. Phys. Rev. B.

[2] Batool Z., Hild K., Hosea T.J.C., Lu X., Tiedje T., Sweeney S.J. (2012). The electronic band structure of GaBiAs/GaAs layers: Influence of strain and band anti-crossing. J. Appl. Phys..

[3] Hossain N., Marko I.P., Jin S.R., Hild K., Sweeney S.J., Lewis R.B., Beaton D.A., Tiedje T. (2012). Recombination mechanisms and band alignment of GaAs_1-x_Bi_x_/GaAs light emitting diodes. Appl. Phys. Lett..

[4] Wang L., Zhang L., Yue L., Liang D., Chen X., Li Y., Lu P., Shao J., Wang S. (2017). Novel dilute bismide, epitaxy, physical properties and device application. Crystals.

[5] Tisbi E., Placidi E., Magri R., Prosposito P., Francini R., Zaganelli A., Cecchi S., Zallo E., Calarco R., Luna E. (2020). Increasing optical efficiency in the telecommunication bands of strain-engineered Ga(As,Bi) alloys. Phys. Rev. Appl..

[6] Fluegel B., Francoeur S., Mascarenhas A., Tixier S., Young E.C., Tiedje T. (2006). Giant spin-orbit bowing in GaAs_1-x_Bi_x_. Phys. Rev. Lett..

[7] Usman M., Broderick C.A., Batool Z., Hild K., Hosea T.J.C., Sweeney S.J., O’Reilly E.P. (2013). Impact of alloy disorder on the band structure of compressively strained GaBi_x_As_1-x_. Phys. Rev. B.

[8] Yastrubchak O., Sadowski J., Gluba L., Domagala J.Z., Rawski M., Żuk J., Kulik M., Andrearczyk T., Wosinski T. (2014). Ferromagnetism and the electronic band structure in (Ga,Mn)(Bi,As) epitaxial layers. Appl. Phys. Lett..

[9] Levchenko K., Andrearczyk T., Domagala J.Z., Wosinski T., Figielski T., Sadowski J. (2015). Impact of bismuth incorporation into (Ga,Mn)As thin films on their structural and magnetic properties. Phys. Status Solidi C.

[10] Levchenko K., Andrearczyk T., Domagała J.Z., Sadowski J., Kowalczyk L., Szot M., Kuna R., Figielski T., Wosinski T. (2017). Novel quaternary dilute magnetic semiconductor (Ga,Mn)(Bi,As): Magnetic and magneto-transport investigations. J. Supercond. Nov. Magn..

[11] Levchenko K., Prokscha T., Sadowski J., Radelytskyi I., Jakiela R., Trzyna M., Andrearczyk T., Figielski T., Wosinski T. (2019). Evidence for the homogeneous ferromagnetic phase in (Ga,Mn)(Bi,As) epitaxial layers from muon spin relaxation spectroscopy. Sci. Rep..

[12] Rodriguez G.V., Millunchick J.M. (2016). Predictive modeling of low solubility semiconductor alloys. J. Appl. Phys..

[13] Wu M., Luna E., Puustinen J., Guina M., Trampert A. (2014). Formation and phase transformation of Bi-containing QD-like clusters in annealed GaAsBi. Nanotechnology.

[14] Gluba L., Yastrubchak O., Domagala J.Z., Jakiela R., Andrearczyk T., Żuk J., Wosinski T., Sadowski J., Sawicki M. (2018). Band structure evolution and the origin of magnetism in (Ga,Mn)As: From paramagnetic through superparamagnetic to ferromagnetic phase. Phys. Rev. B.

[15] Sadowski J., Domagała J.Z., Bąk-Misiuk J., Koleśnik S., Sawicki M., Świątek K., Kanski J., Ilver L., Ström V. (2000). Structural and magnetic properties of molecular beam epitaxy grown GaMnAs layers. J. Vac. Sci. Technol. B.

[16] Edmonds K.W., Bogusławski P., Wang K.Y., Campion R.P., Novikov S.V., Farley N.R.S., Gallagher B.L., Foxon C.T., Sawicki M., Dietl T. (2004). Mn interstitial diffusion in (Ga,Mn)As. Phys. Rev. Lett..

[17] Kuryliszyn-Kudelska I., Domagała J.Z., Wojtowicz T., Liu X., Łusakowska E., Dobrowolski W., Furdyna J.K. (2004). Effect of Mn interstitials on the lattice parameter of Ga_1−x_Mn_x_As. J. Appl. Phys..

[18] Yastrubchak O., Wosinski T., Gluba L., Andrearczyk T., Domagala J.Z., Żuk J., Sadowski J. (2014). Effect of low-temperature annealing on the electronic- and band-structures of (Ga,Mn)As epitaxial layers. J. Appl. Phys..

[19] Puustinen J., Wu M., Luna E., Schramm A., Laukkanen P., Laitinen M., Sajavaara T., Guina M. (2013). Variation of lattice constant and cluster formation in GaAsBi. J. Appl. Phys..

[20] Yastrubchak O., Wosinski T., Domagała J.Z., Łusakowska E., Figielski T., Pécz B., Tóth A.L. (2004). Misfit strain anisotropy in partially relaxed lattice-mismatched InGaAs/GaAs heterostructures. J. Phys. Condens. Matter.

[21] Welp U., Vlasko-Vlasov V.K., Liu X., Furdyna J.K., Wojtowicz T. (2003). Magnetic domain structure and magnetic anisotropy in Ga_1-x_Mn_x_As. Phys. Rev. Lett..

[22] Sadowski J., Domagala J.Z. (2004). Influence of defects on the lattice constant of GaMnAs. Phys. Rev. B.

[23] Dunstan D.J., Brozel M.R., Stillman G.E. (1996). Stiffness of GaAs. Properties of Gallium Arsenide.

[24] Wang K.-Y., Sawicki M., Edmonds K.W., Campion R.P., Maat S., Foxon C.T., Gallagher B.L., Dietl T. (2005). Spin reorientation transition in single-domain (Ga,Mn)As. Phys. Rev. Lett..

[25] Wosinski T., Andrearczyk T., Figielski T., Wrobel J., Sadowski J. (2013). Domain-wall controlled (Ga,Mn)As nanostructures for spintronic applications. Physica E.

[26] Thevenard L., Largeau L., Mauguin O., Patriarche G., Lemaître A., Vernier N., Ferré J. (2006). Magnetic properties and domain structure of (Ga,Mn)As films with perpendicular anisotropy. Phys. Rev. B.

[27] Daeubler J., Schwaiger S., Glunk M., Tabor M., Schoch W., Sauer R., Limmer W. (2008). GaMnAs on InGaAs templates: Influence of strain on the electronic and magnetic properties. Physica E.

[28] Levchenko K., Andrearczyk T., Domagala J.Z., Sadowski J., Kowalczyk L., Szot M., Figielski T., Wosinski T. (2016). Effect of misfit strain in (Ga,Mn)(Bi,As) epitaxial layers on their magnetic and magneto-transport properties. Acta Phys. Polon. A.

[29] Wang Y., Ruterana P., Kret S., El Kazzi S., Desplanque L., Wallart X. (2013). The source of the threading dislocation in GaSb/GaAs hetero-structures and their propagation mechanism. Appl. Phys. Lett..

[30] Dasilva Y.A.R., Kozak R., Erni R., Rossell M.D. (2017). Structural defects in cubic semiconductors characterized by aberration-corrected scanning transmission electron microscopy. Ultramicroscopy.

[31] Wichrowska K., Wosinski T., Domagala J.Z., Kret S., Chusnutdinow S., Karczewski G. (2020). Structural defects in MBE-grown CdTe-based heterojunctions designed for photovoltaic applications. Semicond. Sci. Technol..

[32] De Gennes P.G., Friedel J. (1958). Anomalies de résistivité dans certains métaux magnetiqués. J. Phys. Chem. Solids.

[33] Von Molnár S., Kasuya T. (1968). Evidence of band conduction and critical scattering in dilute Eu-chalcogenide alloys. Phys. Rev. Lett..

[34] Nagaev E.L. (1996). Magnetoimpurity theory of resistivity and magnetoresistance for degenerate ferromagnetic semiconductors of the LaMnO_3_ type. Phys. Rev. B.

[35] Novák V., Olejnik K., Wunderlich J., Cukr M., Vyborny K., Rushforth A.W., Edmonds K.W., Campion R.P., Gallagher B.L., Sinova J. (2008). Curie point singularity in the temperature derivative of resistivity in (Ga,Mn)As. Phys. Rev. Lett..

[36] Wang M., Marshall R.A., Edmonds K.W., Rushforth A.W., Campion R.P., Gallagher B.L. (2014). Determining Curie temperatures in dilute ferromagnetic semiconductors: High Curie temperature (Ga,Mn)As. Appl. Phys. Lett..

[37] Kwiatkowski A., Gryglas-Borysiewicz M., Juszynski P., Przybytek J., Rawicki M., Sadowski J., Wasik D., Baj M. (2016). Determining Curie temperature of (Ga,Mn)As samples based on electrical transport measurements: Low Curie temperature case. Appl. Phys. Lett..

[38] Yuldashev S.U., Yunusov Z.A., Kwon Y.H., Lee S.H., Ahuja R., Kang T.W. (2017). Critical behavior of the resistivity of GaMnAs near the Curie temperature. Solid State Commun..

[39] McGuire T.R., Potter R.I. (1975). Anisotropic magnetoresistance in ferromagnetic 3*d* alloys. IEEE Trans. Magn..

[40] Tang H.X., Kawakami R.K., Awschalom D.D., Roukes M.L. (2003). Giant planar Hall effect in epitaxial (Ga,Mn)As devices. Phys. Rev. Lett..

[41] Matsukura F., Sawicki M., Dietl T., Chiba D., Ohno H. (2004). Magnetotransport properties of metallic (Ga,Mn)As films with compressive and tensile strain. Physica E.

[42] Pelya O., Wosinski T., Figielski T., Makosa A., Morawski A., Sadowski J., Dobrowolski W., Szymczak R., Wróbel J., Tóth A.L. (2006). Magneto-conductance through submicron constriction in ferromagnetic (Ga,Mn)As film. J. Alloys Compd..

[43] Manipatruni S., Nikonov D.E., Lin C.-C., Gosavi T.A., Liu H., Prasad B., Huang Y.-L., Bonturim E., Ramesh R., Young I.A. (2019). Scalable energy-efficient magnetoelectric spin-orbit logic. Nature.

[44] Wang K.-Y., Edmonds K.W., Campion R.P., Zhao L.X., Foxon C.T., Gallagher B.L. (2005). Anisotropic magnetoresistance and magnetic anisotropy in high-quality (Ga,Mn)As films. Phys. Rev. B.

