# Supplementary material for: Structural Quality and Magnetotransport Properties of Epitaxial Layers of the (Ga,Mn)(Bi,As) Dilute Magnetic Semiconductor
Source: Materials (Basel). 2020 Dec 3;13(23):5507. doi: 10.3390/ma13235507 (PMC7730930; doi:10.3390/ma13235507)
Supplement: Supplementary file 1 [file materials-13-05507-s001.pdf]

materials

MDPI

Supplementary Materials: Structural Quality and Magnetotransport Properties of Epitaxial Layers of the (Ga,Mn)(Bi,As) Dilute Magnetic Semiconductor

Tomasz Andrearczyk [1], Khrystyna Levchenko [1,2], Janusz Sadowski [1,3], Jaroslaw Z. Domagala [1], Anna Kaleta [1], Piotr Dłużewski [1], Jerzy Wróbel [1], Tadeusz Figielski [1] and Tadeusz Wosinski [1,*]

[1] Institute of Physics, Polish Academy of Sciences, Aleja Lotnikow 32/46, PL-02668 Warsaw, Poland; andrea@ifpan.edu.pl (T.A.); khrystyna.levchenko@univie.ac.at (K.L.); sadow@ifpan.edu.pl (J.S.); domag@ifpan.edu.pl (J.Z.D.); kaleta@ifpan.edu.pl (A.K.); dluzew@ifpan.edu.pl (P.D.); wrobel@ifpan.edu.pl (J.W.); figiel@ifpan.edu.pl (T.F.)

[2] Faculty of Physics, University of Vienna, 1090 Vienna, Austria

[3] Department of Physics and Electrical Engineering, Linnaeus University, SE-391 82 Kalmar, Sweden

* Correspondence: wosin@ifpan.edu.pl

Received: 30 October 2020; Accepted: 26 November 2020; Published: 30 November 2020

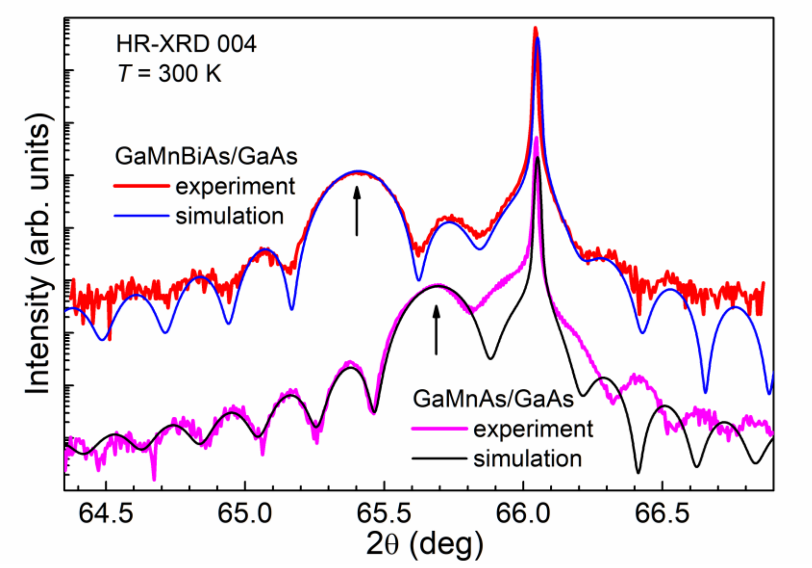

Figure S1. High-resolution X-ray diffraction spectra ($2\theta/\omega$ scans) for the 004 Bragg reflection compared with the simulation results for (Ga,Mn)As and (Ga,Mn)(Bi,As) DMS layers epitaxially grown on (001) GaAs substrate. The narrow, sharp peaks correspond to the GaAs substrate and the broader ones at lower diffraction angles, indicated by the vertical arrows, are signals from the DMS layers. The spectra are vertically offset for clarity. The simulated curves were obtained by fitting the Mn content and the DMS layer thickness, using the EPITAXY program (PANalytical commercial software) based on X-ray dynamic diffraction theory. The best fit values are: 6.1% Mn content and 50 nm thickness for the (Ga,Mn)As/GaAs sample and simulated 10.7% Mn content and 47 nm thickness for the (Ga,Mn)(Bi,As)/GaAs sample (here the simulated 10.7% Mn content stands for the lattice parameter increase in the quaternary alloy caused by both Mn and Bi and corresponds to the real contents of about 6% Mn and 1% Bi).
